# Effects of acute alcohol consumption on hostile attribution bias towards emotional facial expressions and implicit approach/avoidance tendencies

**DOI:** 10.1177/02698811251408743

**Published:** 2026-03-23

**Authors:** Andrew P. R. Eastwood, Ian S. Penton-Voak, Marcus R. Munafò, Angela S. Attwood

**Affiliations:** 1 Psychology, School of Social Sciences, UWE Bristol, UK; 2 School of Psychological Science, University of Bristol, UK; 3 National Institute for Health Research Bristol Biomedical Research Centre, University Hospitals Bristol NHS Foundation Trust and University of Bristol, UK; 4 MRC Integrative Epidemiology Unit, University of Bristol, UK

**Keywords:** hostile attribution bias, emotional facial expressions, acute alcohol consumption, approach/avoidance tendency, alcohol related aggression

## Abstract

**Background:**

Research suggests that a greater perception of hostility in social cues increases aggression, and alcohol influences perception of social cues. Taken together, this could explain some instances of alcohol-related aggression. This study investigated whether social drinkers interpret faces as more hostile following acute alcohol compared to placebo, and whether alcohol influences the tendency to approach or avoid emotional facial expressions.

**Methods:**

Regular non-dependent drinkers (*N* = 84) participated in a double-blind placebo-controlled experiment. Participants completed two sessions and were tested following an alcoholic drink (0.4 g/kg), and matched placebo. In each session, they completed tasks measuring hostile attribution bias (HAB) towards emotional faces (happy, sad, angry, disgust, surprise, and fear), and approach/avoidance tendencies towards emotional faces (angry, happy, sad and disgust).

**Results:**

Alcohol did not affect global hostility ratings of emotional facial expression (*p* = 0.342). However, it did increase global hostility ratings of ambiguous emotional faces after alcohol (drink by intensity interaction; *p* = 0.002). At an emotion-specific level, happy faces were seen as more hostile after alcohol when compared to placebo (*p* = 0.009; irrespective of emotional intensity). Alcohol did not affect approach/avoidance tendencies when seeing emotional faces following alcohol.

**Conclusions:**

These findings suggest that alcohol increases hostile judgements of ambiguous emotional faces. They also suggest that happy faces are perceived to be more hostile following alcohol. As an increased HAB when processing socially relevant information increases aggressive responding, this increased hostile perception of happy faces following alcohol may increase the likelihood of aggressive behaviour.

## Introduction

Emotional facial expressions are considered to be a fundamental component of effective social interaction (Moriya et al., 2013), which are capable of influencing behaviour. Research has demonstrated that acute alcohol consumption influences the perception of these emotional expressions. Our previous work suggests poorer emotion recognition following an acute dose of alcohol compared to placebo. At an emotion-specific level, the ability to see sadness and fear is impaired (Eastwood et al., 2020). Attwood et al. (2009) reported an increased bias towards seeing anger in ambiguous facial morphs following acute alcohol consumption. This anger bias has been replicated in a more recent study, although effect sizes are small (Khouja et al., 2019). The reduced ability to see expressions associated with submissive behaviour (i.e. sadness and fearful expressions) paired with an anger perception bias (albeit small) may function to increase aggressive responding. The tendency to perceive or interpret others behaviour as hostile is often referred to as hostile attribution bias (HAB; Nasby et al., 1980). Research suggests that higher levels of this bias are associated with increased aggression (Chen et al., 2012; Crick et al., 2002; Dodge, 2006). This can have negative social consequences, as perceived aggressive intent plays a causal role in reactive aggressive behaviour (Crick and Dodge, 1996).

Within the literature, an increased bias towards seeing anger has been interpreted as an increased bias towards judging facial expressions as hostile (Wegrzyn et al., 2017). However, ‘anger’ and ‘hostility’ conceptually differ (Eckhardt et al., 2004). Anger is an emotion most associated with feelings of irritation, annoyance, fury, and rage. State-anger is often described as the response to an emotional elicitor that induces these feelings, whilst trait-anger is thought to be a more constant personality trait characterised by more frequent experiences of these feelings even when the cues are innocuous or unprovocative (Ramírez and Andreu, 2006).Hostility, on the other hand, can be considered to be an individual attitude that involves negative evaluations of others (Eckhardt et al., 2004). The perception of hostility communicates the intention to harm an individual, including expressive characteristics that signal intent for physical violence (Deffenbacher, 2000). In support of a difference between anger and hostility, one study found that facial displays of hostility produced greater physiological arousal than displays of anger (Tsikandilakis et al., 2020). Hostile interpretations may not be restricted to angry faces. It is likely that other emotions, or emotionally ambiguous facial expressions, may also be interpreted as hostile. For example, a disgusted face in particular may be judged as more hostile as it shares similar expressive characteristics to anger (Wieser and Brosch, 2012). In addition, the unique characteristics of an emotional face, like disgust, for example, may also contribute to hostile interpretations of these expressions (i.e. not just sharing similar expressive characteristics with anger, but displaying hostile expressive features in their own right). Recent research has investigated HAB in facial affect using a sample of typically aggressive individuals (i.e. forensic outpatient population; Smeijers et al., 2017). This research presented individuals with images of four facial expressions of emotion (angry, fear, disgust, and happy), which were judged as either displaying hostility or not. They found that individuals with an aggression regulation deficit (i.e. antisocial and borderline personality disorder) demonstrate an increased perception of hostility in emotional expressions (angry, disgusted, fearful and happy faces) compared to healthy controls. The authors discuss this HAB towards emotional stimuli, as a key characteristic of pathological aggression in a forensic outpatient sample. To our knowledge, this is the first study to investigate HAB of emotional stimuli by treating angry expressions and hostile judgements as separate concepts. These hostile judgements are also likely to be more pronounced in ambiguous displays of emotional expressions. Prior research suggests that ambiguous emotional expressions (i.e. those that are not clearly identifiable as a specific emotion) are more likely to be interpreted as hostile, particularly amongst typically aggressive groups (Schonenberg and Jusyte, 2014; Wegrzyn et al., 2017). This is a particularly important factor to consider given that alcohol impairs emotion recognition (Eastwood et al., 2020; Khouja et al., 2019), by increasing the ambiguity of the perceived emotional expressions, which in turn might increase hostile judgements of these emotions. It is therefore anticipated that alcohol will increase hostile judgements of emotions, and increased ambiguity of the facial expression will increase the likelihood of these judgements.

Another potential contributing factor in alcohol-induced aggression surrounds the behavioural response (i.e. whether to approach or avoid) when perceiving emotional facial expressions. Evidence typically suggests that we tend to approach positive stimuli and avoid negative stimuli (Chen and Bargh, 1999). While positive stimuli typically elicit approach tendencies, there is evidence in the context of aggression that negative stimuli can also trigger approach tendencies (Harmon-Jones and Schutter, 2022). In the facial affect literature, findings are mixed. One argument is that individuals tend to automatically avoid potentially threatening situations by approaching happy faces (Seidel et al., 2010), whilst avoiding angry faces (Heuer et al., 2007; Marsh et al., 2005). However, Veenstra et al. (2017) argue that individuals high in trait aggression demonstrate quicker approach responses to angry faces. These findings could be attributed to increased testosterone levels associated with aggressive tendencies (Batrinos, 2012), biasing individuals to approach a perceived surmountable social threat (Enter et al., 2014). This was further supported by Bossuyt et al. (2014), who concluded that approach/avoidance behaviour was goal-dependent; they found that angry faces were approached if the manipulated goal was to dominate/aggress. With regards to the alcohol literature, there is little research investigating approach/avoidance tendencies towards emotionally expressive facial stimuli under the influence of alcohol. Some evidence suggests that acute alcohol consumption increases testosterone levels when administered in low doses (Sarkola and Eriksson, 2003; Sarkola et al., 2000). It is therefore anticipated that hostile cues (i.e. angry and disgusted faces) are less likely to be avoided and more likely to be approached following alcohol compared to placebo.

The aims of this research were to investigate the effect of acute alcohol consumption on two primary outcomes. The first was HAB of emotional facial expressions (happy, sad, anger, disgust, surprise, fear) and the second was approach/avoidance tendencies towards emotional facial expressions (angry, happy, sad, disgust). An adapted version of the Hostility Interpretation Bias Task (HIBT) developed by Smeijers et al. (2017) was used to measure HAB in the study. Participants categorised composite images of happy, sad, angry, disgusted, surprised, and fearful male faces as either hostile or not hostile. It was hypothesised that there would be greater HAB towards emotional facial expressions (i.e. increased percentage of hostile judgements) following acute alcohol consumption compared to placebo. In addition, emotion-specific HAB (i.e. % of hostile judgements) was also explored following alcohol for each emotion (happy, sad, anger, disgust, surprise, fear). Emotional intensity was also considered as an exploratory factor due to its potential influence on HAB. It was anticipated that the ambiguity of the target emotional facial expression would influence hostile interpretations following alcohol consumption compared to placebo. The emotional intensity displayed in each face ranged from ambiguous to full example along a 15-image continuum (consistent with previous facial expression research, Eastwood et al., 2020). It was hypothesised that ambiguous displays of emotions would be interpreted as more hostile following alcohol, when compared to placebo. And this effect would diminish as emotional intensity increases. Past research indicated greater HAB in individuals who were typically aggressive (Smeijers et al., 2017) and demonstrated problematic drinking behaviour (Frigerio et al., 2002). To isolate the effect of alcohol consumption on HAB towards emotional facial stimuli, exploratory demographic variables of age, gender, trait anger, and alcohol use were statistically controlled. For approach avoidance tendencies, it was hypothesised that there would be less avoidance of hostile emotions (i.e. angry and disgusted) (i.e. faster reaction times (RTs) in approach trials compared to avoidance) following acute alcohol compared to placebo. In addition, approach/avoidance tendencies (i.e. RTs) towards non-hostile emotions (i.e. happy and sad faces) were also explored following acute alcohol, compared to placebo.

## Methods

### Participants

Social drinkers (*N* = 84, 50% male) were recruited from the University of Bristol (staff and students) as well as the general population by means of existing email lists, poster advertisement, and word of mouth. Eligibility was confirmed by the researcher using a structured inclusion/exclusion checklist during screening. The inclusion criteria included good physical and psychiatric health, aged between 18 to 40 years and speaking English as first language or equivalent level of fluency. The age of participants was capped at 40 to minimise variability in cognitive processes associated with ageing, such as changes in emotion recognition (i.e. reduced accuracy in identifying negative emotions, especially when these emotions are displayed at low intensity; Durbin et al., 2020). To avoid including participants with little/no drinking experience or undiagnosed alcohol dependence, only individuals who consumed between 5 and 35 alcoholic units per week were included. One UK unit equals one 25 ml single measure of spirit (Alcohol by Volume [ABV] 40%), or a third of a pint of beer (ABV 5%–6%) or half a standard (175 ml) glass of red wine (ABV 12%) (NHS UK, 2018). The exclusion criteria were any individuals that reported a strong familial history of alcoholism (in parents and/or siblings) or that reported a history of psychiatric disorder (including drug addiction), alcohol consumption within 24 hours prior to testing or if their breath alcohol concentration (BrAC) was above zero (tested on arrival), and if they weighed less than 50 kg if female or 60 kg if male. Participants gave signed informed consent prior to taking part in the study. Participants were reimbursed £15 on completion of the study or were awarded equivalent course credits. The study was approved by the University of Bristol’s Faculty of Science Human Research Ethics Committee (reference: 25011860401). The study protocol was pre-registered on the Open Science Framework (doi: 10.17605/OSF.IO/2EN6M).

Sample size was determined from an effect size obtained in our previous study that investigated the effects of an acute dose of alcohol on emotional facial expression processing, in high versus low trait aggressive drinkers (Eastwood et al., 2020). This study used a 6-alternative forced choice task to investigate global emotion processing accuracy, using total hit rate as the primary outcome measure, and indicated that alcohol administration resulted in lower emotion processing accuracy (*M* = 99.8, SD = 12.6) compared to placebo (*M* = 103.4, SD = 12.4). This suggested an effect size of dz = 0.36 (correlation between conditions *r* = 0.68). Based on these data, we would need a total sample size of 84 participants to achieve 90% power (alpha level of 5%) to observe an effect of alcohol on hostile attributions of a similar size. The present study includes a more complex design with multiple exploratory conditions and outcomes. The power analysis was specifically intended to ensure sufficient sensitivity to detect a main effect of alcohol on HAB, which was conceptually and methodologically analogous to the primary outcome in the prior study (i.e. a within-subject comparison of responses under alcohol vs. placebo). Other effects, including interactions, were considered exploratory.

### Design

A double-blind placebo-controlled experimental design with one within-subject factor of drink (0.4 g/kg alcohol, placebo) was used. The primary dependent measure of the Hostile Attribution Bias Task (HABT) was percentage of ‘hostile’ responses. For this measure, an additional within-subjects factor of target emotion was used (happy, sad, angry, disgust, surprise and fear). The primary dependent measure of the approach avoidance task (AAT) was a RT bias score (i.e. approach RT scores subtracted from avoidance RT scores). This measure included an additional within-subjects factor of emotion (happy, angry, sad, and disgust) and target face ethnicity (white, black). Cunningham et al. (2012) show that people rapidly and automatically categorise faces by race, which can influence early perceptual processes therefore, target face ethnicity was included as an exploratory variable in order to determine whether this influenced approach/avoidance tendencies. Error rates (i.e. proportion of errors) were also recorded to control for their influence on approach/avoidance RTs. Session order was counterbalanced with equal numbers of participants in each order group. Participants were allocated session orders in advance of the study using random number generator software (www.randomizer.org).

### Materials

#### Hostile attribution bias task

**Figure 1.**
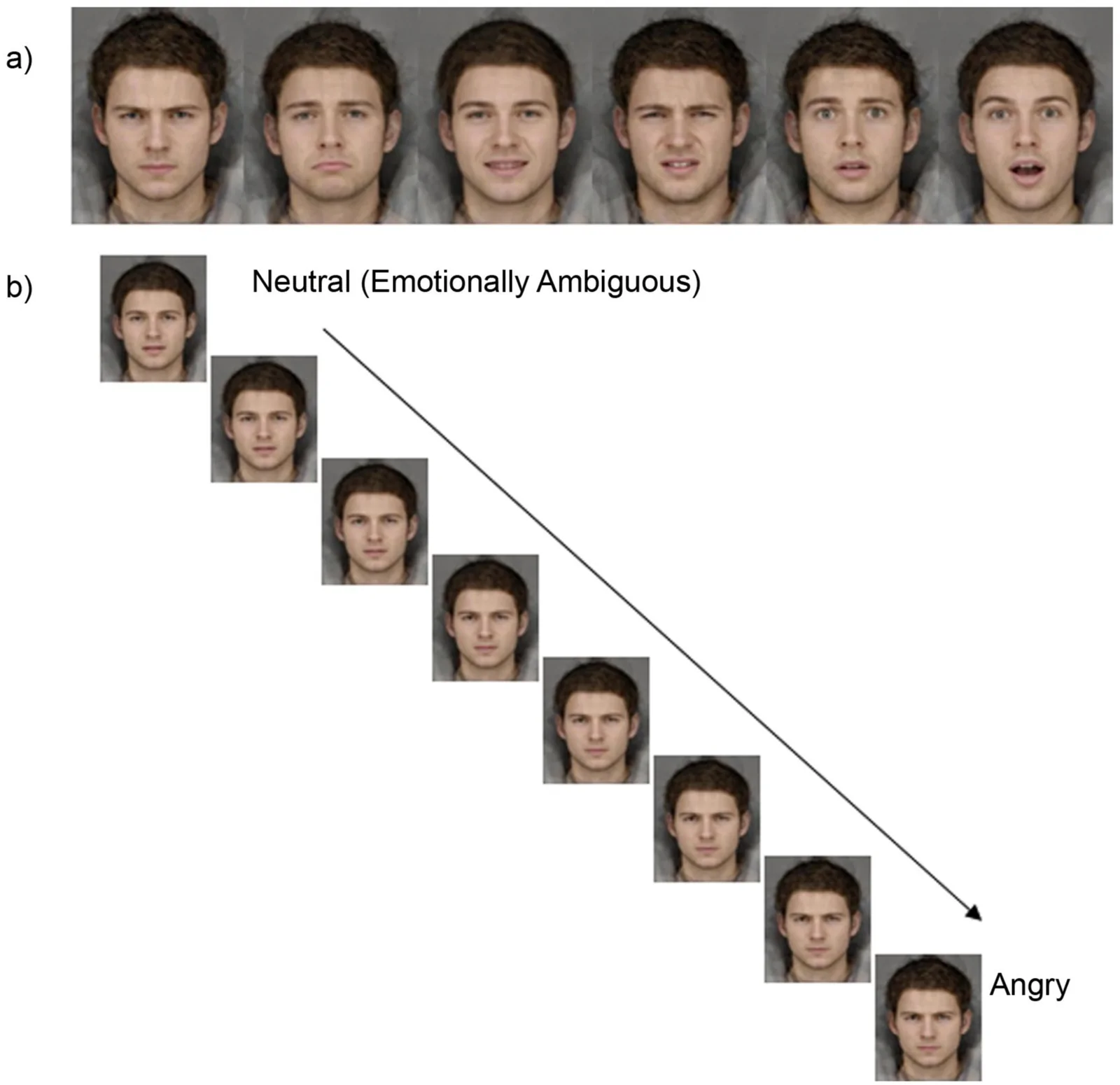
(a) Full intensity examples of the six basic emotions used in the HABT task. Facial expressions are angry, sad, happy, disgust, fear, surprise from left to right. (b) 15-image morph sequence for the angry emotion. Stimuli range from emotionally ambiguous to full emotion intensity. HABT: Hostile Attribution Bias Task.

The HABT was an adapted version of the HIBT developed by Smeijers et al. (2017). The images used in the HABT were composite (i.e. prototypical) faces created from photographs of 12 young male adults photographed under controlled conditions. These photographs were taken in a booth painted Munsell N5 grey and illuminated with three Verivide F20 T12/D65 daylight simulation bulbs in high-frequency fixtures (Verivide, UK) to minimise flicker effects. Using established techniques (Tiddeman et al., 2001), each image was delineated with 172 feature points, allowing colour and shape information to be averaged across faces to produce a full prototypical exemplar expression for each of 6 emotions: anger, sadness, happiness, disgust, fear, and surprise (see Figure 1(a)).

For each emotion, 15 images were generated along a linear continuum, ranging from an emotionally ambiguous prototype (5% target emotion) to the highest level of emotional intensity (100% target emotion) in equal increments. This ensured that every image contained at least a nominal emotional signal, allowing categorisation accuracy to be assessed across all stimuli. In practice, the starting image in each continuum appeared fully ambiguous despite containing a very small emotional signal. These stimuli have been used in previous research (Eastwood et al., 2020). Figure 1(b) illustrates the prototypical emotions and an example morph continuum.

Each trial began with a centrally displayed fixation cross, followed by a 350 × 457-pixel face stimulus presented for 150 mseconds, then a noise mask for 250 mseconds to prevent after-image effects. A presentation duration of 150 mseconds was selected because previous research demonstrates that impressions from faces can be formed rapidly. Judgements made after only 100 mseconds strongly correlate with those obtained under unconstrained viewing conditions, with additional exposure primarily enhancing confidence rather than altering the nature of the judgement (Willis and Todorov, 2006). Consequently, a 150 mseconds exposure is sufficient to capture rapid, automatic evaluative processes while minimising the influence of strategic or memory-based processing. The HABT was implemented in E-Prime 2.0 Pro software and run on a standard computer with a QWERTY keyboard. On each trial, 1 image from the 90 available (6 emotions × 15 intensities) was presented for 150 mseconds and backward masked by visual noise. Labels reading ‘Hostile’ and ‘Not Hostile’ (Arial, size 30) were displayed at the bottom left and bottom right of the screen, respectively. Each image was presented twice, resulting in 180 trials in total. Participants responded by pressing the ‘c’ key for ‘hostile’ or the ‘m’ key for ‘not hostile’ as quickly as possible. The primary outcome measure was the percentage of hostile responses, with higher values reflecting a stronger perception of hostility in the emotional stimulus presented.

#### Approach avoidance task

This task used similar composite face stimuli as described in the HABT, but only full-intensity expressions of anger, happiness, sadness, and disgust were used. Participants first completed a practice block of 12 trials featuring neutral facial expressions, followed by 2 experimental blocks of 64 trials each (128 trials total). Each experimental block included 32 unique images: angry, happy, sad, and disgusted faces from both white and black ethnicities, each repeated 4 times. Every image was presented in both an approach and an avoidance trial, resulting in 64 trials per block. Each trial began with a fixation cross displayed for 500 mseconds, followed by a face stimulus. After a brief delay (500–750 mseconds), the image was framed either by a solid line or a dashed black line. A solid line frame cued the participant to approach by pulling the image towards them (arm flexion; 50% of trials), using a computer joystick and doing so increased the size of the image. A dashed black frame cued the participant to avoid by pushing the image away from them (arm extension; 50% of trials) and doing so reduced the size of the image (Phaf et al., 2014). This zooming effect provided the participant with feedback on each trial so that the image increasing and decreasing in size reinforced a sense of approach and avoidance, respectively (Phaf et al., 2014). The stimulus remained on the screen for 10,000 mseconds and participants were encouraged to respond as quickly and as accurately as possible. Trials ended if no response was made within this timeframe. Stimulus presentation order was randomised within blocks and across participants.

The primary outcome variables were approach and avoidance RTs. Each RT was measured from the presentation of the cue to the disappearance of the image following a response in the approach and avoidance trials. These RTs are interpreted as indicators of the strength of automatic motivational tendencies: faster responses suggest a stronger preference for the cued action (e.g. a fast approach response reflects a strong tendency to approach), whereas slower responses may indicate hesitation or weaker alignment with the cue. Error rates were calculated as the proportion of incorrect responses, such as making an approach response when avoidance was cued, or vice versa. These errors are interpreted as reflecting a mismatch between the participant’s automatic tendency and the instructed action. For example, a high error rate in approach trials suggests that the participant had a stronger underlying tendency to avoid the stimulus. Conversely, low error rates indicate greater consistency between the participant’s response and the cue, suggesting alignment with the intended motivational direction. Error scores were systematically controlled for during analysis to ensure accurate interpretation of RT data.

#### Questionnaires

Questionnaires used were the Alcohol Use Disorders Identification Test (AUDIT) (Saunders et al., 1993); higher scores on this measure indicate more problematic drinking behaviour consumption. The State anger (S-Ang) and Trait anger (T-Ang) subscales of the State-Trait Anger Expression Inventory (STAXI-2; Spielberger, 1999); higher scores on each subscale represent great aggressive tendencies. The Positive and Negative Affect Schedule (PANAS; Watson et al., 1988); higher scores represent greater positive/negative affect.

### Procedures

Eligible participants were required to attend 2 testing sessions (approximately 60 minutes each), scheduled at least 7 days apart. In session one, participants were given the opportunity to read the information sheet again and ask questions, before providing written informed consent. The researcher then conducted a short screening procedure to verify eligibility, which included measures of weight and an alcohol breath test (Draeger AlcoDigital 3000 Breathalyser) to confirm zero BrAC. Weight information was passed to a research collaborator for drink preparation. Participants then completed baseline questionnaire measures (pre-consumption), including the AUDIT, STAXI-2, and PANAS. During both testing sessions, participants received a single drink to consume. In session one, this contained either 0.4 g/kg alcohol or a matched placebo. Drinks were prepared by a research collaborator who was independent of data collection and drink administration was double-blind. Alcohol content was dependent on participant weight. An upper limit of 90 kg was set so that participants weighing more than 90 kg received the same drink as a 90 kg participant. The alcoholic drinks were mixed using one-part vodka (37.5% ABV) to three parts tonic water. The dose used was 0.4 g of alcohol per kg of body weight (g/kg) (Attwood et al., 2009; Craig et al., 2009). Placebo drinks were matched volume tonic water. In order to mask the taste of alcohol, drinks were chilled and flavoured with lime cordial (40 ml) prior to serving. The inside rim of the glass was sprayed twice with a vodka mist. The opposite drink was administered in session two (order counterbalanced). Participants were given 10 minutes to consume all of their drink and a further 10 minutes to sit quietly to allow for absorption. Next, participants completed the HABT and the AAT (fixed order) and each task took approximately 12 to 15 minutes to complete. They then completed the questionnaire measures a second time post-consumption (STAXI-2; S-Ang Subscale, PANAS), and a second BrAC reading. The timing of this procedure was designed to capture participants during the ascending limb of the BrAC curve to measure acute alcohol effects (Schweizer et al., 2006). After consuming the 0.4 g/kg dose within 10 minutes, participants were given a 10-minute absorption period to allow partial uptake of alcohol into the bloodstream. At this point (approximately 20 minutes post-ingestion), BrAC is typically still rising and has not yet reached peak levels, which generally occur 30 to 60 minutes after consumption. The subsequent HABT and AAT (lasting approximately 30 minutes in total) were scheduled so that participants would complete them while BrAC was increasing or near its peak, ensuring that task performance reflects the pharmacological effects associated with the ascending limb rather than the descending limb, where tolerance and acute adaptation may occur (Holland and Ferner, 2017). Participants received standardised, scripted verbal instructions throughout the testing sessions to ensure procedural consistency. These instructions covered all key stages of the study, including questionnaire completion, drink consumption, and computer-based tasks (HABT and AAT). Before leaving the session, participants were required to read and sign a safety card and were offered the opportunity to stay behind until they feel any effects of alcohol have worn off. They were also offered a taxi home if required. At the end of session two, participants were debriefed and reimbursed.

### Statistical analysis

Statistical analyses were conducted using R Studio (2019; R Core Team, 2020). For the HABT data, an error in programming the task meant that the presentation of the surprise emotion was compromised. This error meant that some of the participants were presented with 2 full intensity surprise images and 28 emotionally ambiguous images (i.e. 5% along the continuum between ‘emotional ambiguity’ to ‘full intensity’ surprise) when completing the task. As a result, the erroneous surprise data was removed from analysis. It was originally planned that this data would be analysed using 2 drink (alcohol, placebo) × 5 emotion (happy, sad, angry, fear, disgust) repeated measures ANOVA with interactions being explored using t-tests. However, it was later decided that this data would be analysed using linear mixed effects (LME) modelling (Baayen et al., 2008). This allows for the systematic control over the random between-subject’s variance whilst controlling for other fixed effect variance (age, gender, trait anger, AUDIT). It also allows for the exploration of the interaction between emotion intensity (i.e. [1] emotionally ambiguous – [15] full emotionally intensity) and Drink (alcohol, placebo). Multivariate normality, homoscedasticity and multicollinearity assumptions were satisfied unless otherwise stated. Analyses outlined in the study protocol are reported in *Supplemental Text S1* . To evaluate whether participant mood (i.e. positive/negative affect) or state anger changed across pre- and post-consumption time points for both alcohol and placebo conditions, a series of 2 Drink (alcohol, placebo) × 2 Time (pre-consumption, post-consumption) repeated-measures ANOVAs were used on Positive Affect, Negative Affect, and State Anger scores.

The primary objective of this research was to investigate whether alcohol influences HAB when perceiving emotional facial expressions and this study was powered to detect an effect of drink (i.e. alcohol vs. placebo). To test hostile ratings of emotional facial expressions globally, LME models were used with two blocks (lme4 package in r; Bates et al., 2015). In the first block (*main effects block*), fixed effects of drink and intensity were entered into the LME model to test the main effects of drink and intensity on hostile ratings. In the second block (*interaction block*), a drink by intensity interaction term was entered into the model to test the interaction between drink and emotional intensity on hostility ratings. Age, gender, trait anger and AUDIT scores were also entered as fixed effects to adjust for their influence. Random intercepts for subject ID, as well as by ID random slopes for the effect of drink were entered as random effects. *p* values for each fixed effect were estimated using Kenward-Roger degrees of freedom (Luke, 2017). For emotion-specific analyses of angry, happy, sad, disgust, and fearful faces the same LME models were applied.

For the AAT, three participants were removed from the analysis as error rates were relatively high (three standard deviations from the mean). Further inspection of these data suggests that the participants misunderstood the task (i.e. high errors scores indicated using the task response options the wrong way around). It was planned that this data would be analysed using a 2 drink (alcohol, placebo) × 2 ethnicity (white, black) × 4 emotion (happy, angry, sad, disgust) repeated measures ANOVA to explore RT bias scores (i.e. the difference between approach and avoidance RT scores). It was also planned to analyse error rates using a 2 drink (alcohol, placebo) × 2 ethnicity (white, black) × 4 emotion (happy, angry, sad, disgust) × 2 tendency (approach, avoidance) repeated measures ANOVA. Instead, LME models were used to investigate approach/avoidance tendencies (i.e. approach and avoidance RTs) following alcohol and placebo, for each of the four emotions (happy, angry, sad, disgust). For each emotion, LME models were used with two blocks (lme4 package in r; Bates et al., 2015). In the first block (*main effects block*), drink and tendency were entered into the model as a fixed effect. In the second block (*interaction block*), a drink by tendency interaction term was entered into the model. Error rate was also entered as a fixed effect to control for their influence, as well as age, gender, trait anger, AUDIT scores, face ethnicity. As random effects, we had random intercepts for subject ID, as well as by ID random slopes for the effect of drink. *p* values for each fixed effect were estimated using Kenward-Roger degrees of freedom (Luke, 2017).

## Results

### Participant characteristics

Participants were aged between 18 to 40 years (*M* = 22.5, SD = 4.4) and weighed between 50 to 117 kg (*M* = 71.2, SD = 13.0). Scores on the AUDIT ranged from 3 to 34 (*M* = 10.3, SD = 4.9) and scores on the trait aggressive subscale of the STAXI-2 ranged from 10 to 28 (*M* = 15.6, SD = 3.7). Post session BrAC in the alcohol condition ranged from 8 to 32 µg/100 ml (*M* = 16.62, SD = 4.81). For reference, the UK legal driving limit for BrAC is 35 µg of alcohol per 100 ml of breath (Langford and Ferner, 2013). Positive and negative affect, and state anger scores can be seen in Table 1. A series of repeated-measures ANOVAs was conducted to examine changes in positive affect, negative affect, and state anger. For positive affect, there was a significant main effect of time, *F*(1, 83) = 37.60, *p* < 0.001, η²G = 0.04, and a significant drink × time interaction, *F*(1, 83) = 5.93, *p* = 0.017, η²G = 0.004. Scores decreased over time for both drinks, with a larger reduction following the placebo than alcohol. There was no main effect of drink, *p* > 0.965. For negative affect, there was a significant main effect of time, *F*(1, 83) = 20.97, *p* < 0.001, η²G = 0.04, indicating a reduction from pre- to post-consumption across both drink conditions. No main effect of drink or drink × time interaction was observed (ps > 0.295). For state anger, no significant main effects or interactions were found, indicating that anger levels remained stable across time and drink conditions (ps > 0.084). While positive and negative affect showed decreases over time, these changes were relatively consistent across drink conditions, and state anger remained stable. The absence of systematic changes in mood or anger attributable to the drink condition suggests that these variables are unlikely to have confounded the main effects observed in the primary analyses. When asked on completion of each testing session, 83.3% of participants believed they had consumed alcohol when the drink administered was alcohol. In comparison, 40.5% believed they had consumed alcohol when the drink administered was a placebo.

**Table 1.** Mean and SD for positive/negative affect and state anger scores pre and post drink consumption in the alcohol and placebo conditions.

Drink	Time	Positive affect	Negative affect	State anger
Alcohol	Pre	25.26 (7.43)	13.23 (4.71)	16.33 (3.17)
	Post	23.36 (7.95)	11.52 (2.67)	16.25 (4.04)
Placebo	Pre	26.21 (6.76)	13.23 (4.15)	15.73 (1.65)
	Post	22.45 (7.72)	11.99 (3.69)	15.80 (1.70)

*N* = 84. Positive and Negative Affect measured using PANAS; State Anger measured using STAXI-2.

SD: Standard deviation; PANAS: Positive and Negative Affect Schedule.

### HAB of emotional stimuli

#### Global emotion hostile ratings

Mean percentages of hostility ratings for global score are presented in Table 2, with full model estimates shown in Supplemental Table S1. The primary research objective was to test whether alcohol increased hostile judgements of emotional facial expressions when compared to placebo, and results show no significant main effect of drink condition on global HAB ratings (*p* = 0.342). This indicates that alcohol did not significantly impact overall hostile judgements of emotional faces. As part of the exploratory objectives, analyses examined whether emotional intensity and its interaction with drink condition influenced HAB. There was a significant main effect of emotional intensity (*p* < 0.001), with hostility ratings increasing by approximately 1.61% for each incremental increase in intensity. Exploratory analyses showed a significant drink × intensity interaction (*p* = 0.002), such that alcohol was associated with a 3.48% increase in hostility ratings at intensity level 0 (emotionally ambiguous). Under alcohol, hostility ratings increased by 1.46% per intensity step, whereas under placebo, ratings increased by 1.76% per step (see Figure 3(a)). This suggests that alcohol may amplify hostile interpretations of ambiguous emotional expressions, but the rate of increase in hostility with rising emotional intensity is slightly attenuated under alcohol. The model was adjusted for age, gender, trait anger, and AUDIT scores. Age and AUDIT scores were significantly associated with reduced HAB ratings (*p* = 0.002 and *p* = 0.036, respectively), while gender and trait anger showed no significant effects (ps > 0.564).

**Table 2.** Mean and SD for hostility ratings of emotional facial expression (Angry, happy, sad, disgust, fear) following alcohol and placebo drinks.

Emotion	Alcohol	Placebo
Angry	71.76 (17.67)	72.11 (15.62)
Happy	8.09 (12.87)	5.07 (10.24)
Sad	10.10 (16.40)	8.62 (15.20)
Disgust	63.58 (30.41)	60.74 (29.82)
Fear	4.52 (12.42)	6.03 (16.68)
**Global**	**31.61 (17.95)**	**30.51 (17.91)**

*n* = 84. The boldface values represent global hostility scores represent the mean of hostility ratings across the 5 emotional expressions.

SD: Standard deviation.

#### Emotion-specific hostile ratings

**Figure 2.**
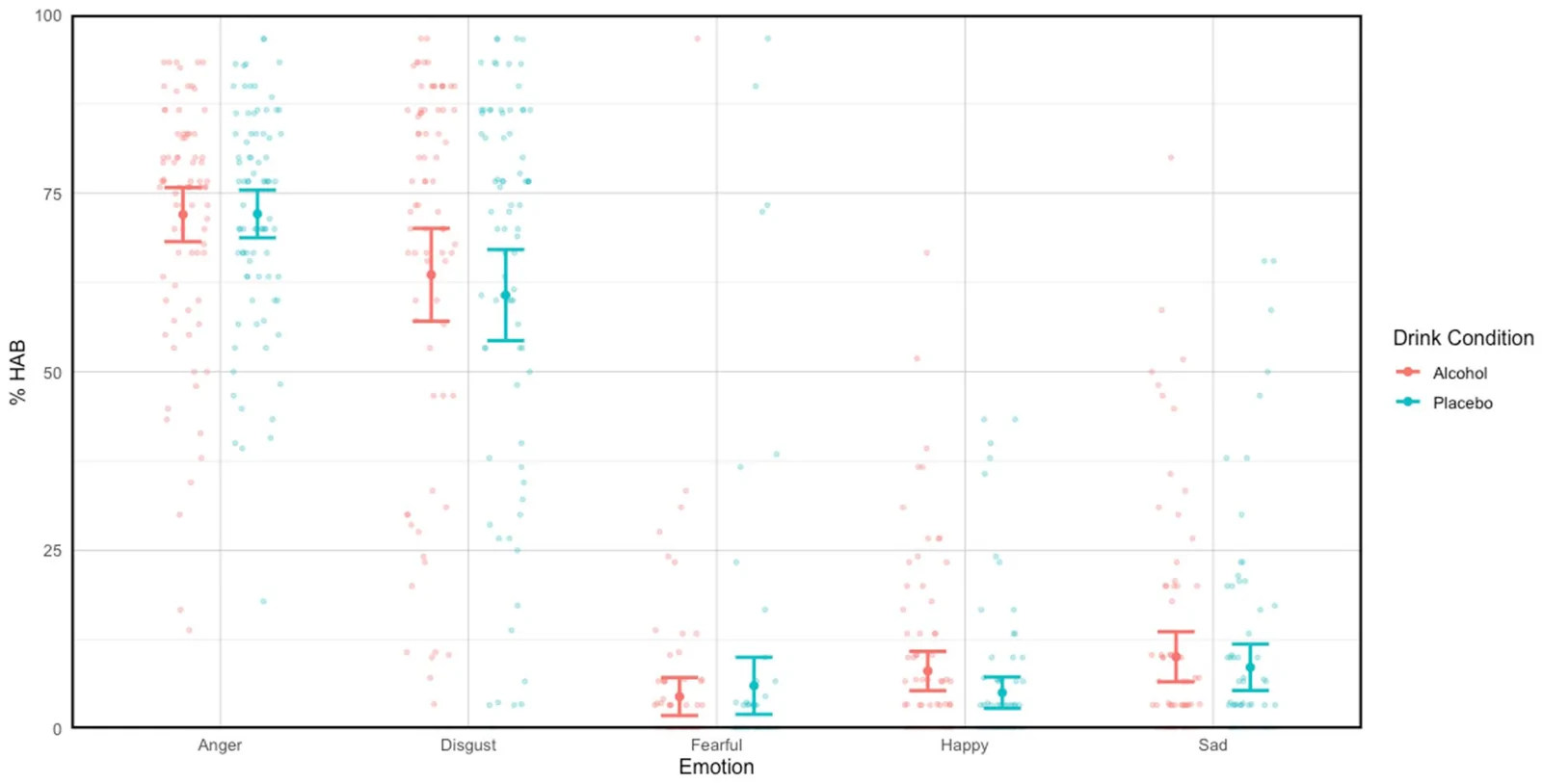
% Hostility rating for each emotional facial expression (Happy, angry, disgust, fear, sad) following alcohol and placebo drinks. Error bars represent 95% confidence intervals.

**Figure 3.**
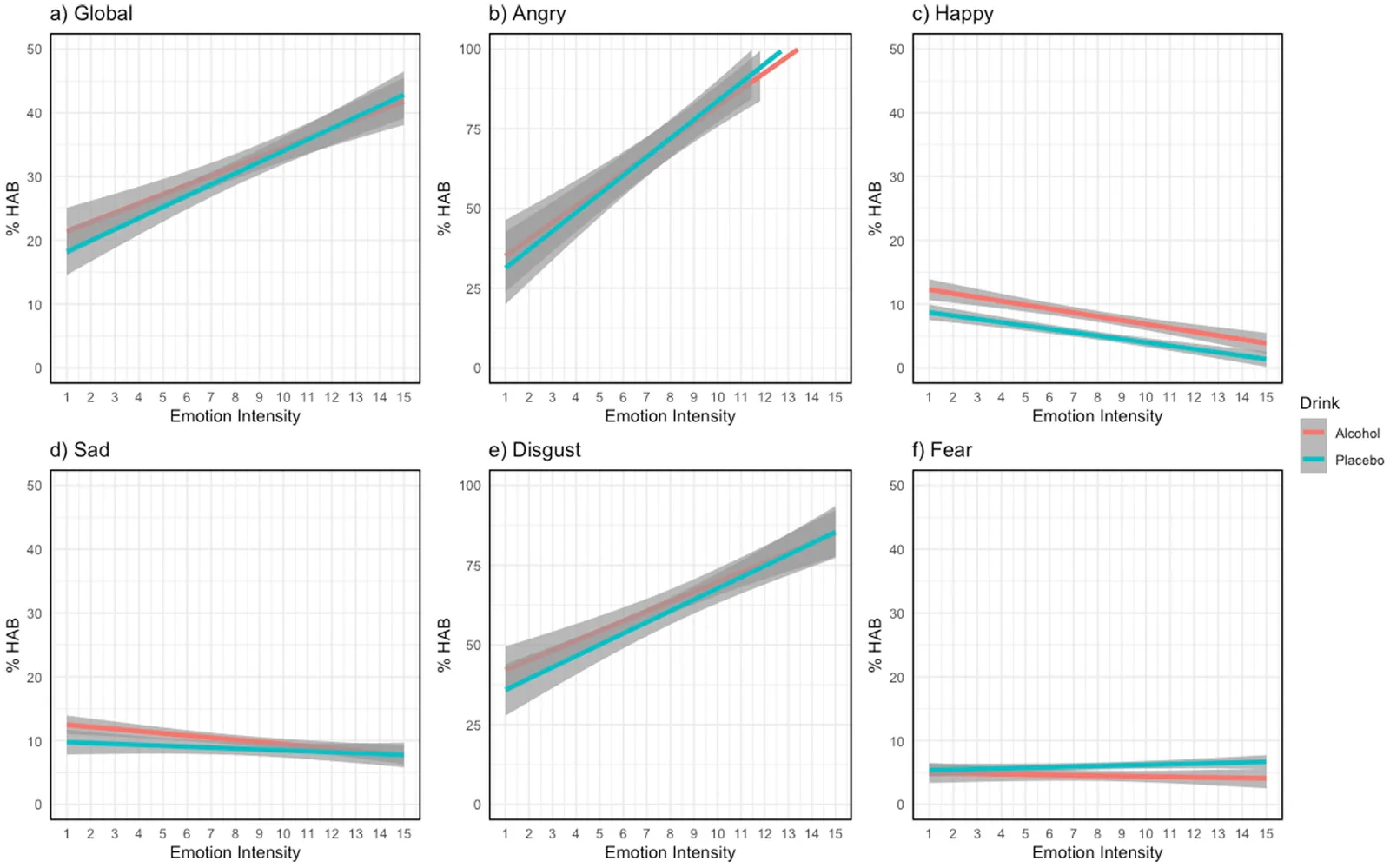
The relationship between % Hostile Rating and emotional intensity (1 – emotionally ambiguous – 15 full example of emotion) following alcohol and placebo drinks. (a) Global hostile ratings of emotional facial expressions, (b) hostile ratings of angry face.

Mean percentages of hostility ratings for each emotion are presented in Table 2, with full model estimates in Supplemental Table S1. The primary objective was to test whether HAB scores varied by emotion (happy, sad, anger, disgust, surprise, fear) following alcohol consumption, when compared to placebo. In addition, exploratory analyses examined whether these effects were influenced by emotional intensity. There was a significant main effect of drink condition for happy expressions, with hostility ratings approximately 3.01% higher following alcohol compared to placebo (*p* = 0.009; see Figure 2). There was no evidence for a drink effect on hostile ratings of angry, sad, disgusted, or fearful expressions (ps > 0.210), suggesting that alcohol selectively increased hostile judgements of happy faces. Significant main effects of emotional intensity were observed for angry, happy, and disgusted faces (ps < 0.001). Hostility ratings increased by an estimated 5.51% and 3.30% for every unit increase in intensity for angry and disgusted expressions, respectively, while ratings for happy expressions decreased by approximately 0.56% per unit increase. A smaller effect of intensity was also observed for sad expressions (*p* = 0.006), with hostility ratings decreasing by 0.24% per unit increase. Exploratory analyses showed that a significant drink × intensity interaction was observed for angry expressions (*p* = 0.019; see Figure 3(b)), with alcohol associated with a 4.40% increase in hostility ratings at the lowest intensity level. As anger intensity increased, hostility ratings rose by 5.21% per step following alcohol and 5.81% per step following placebo. No significant drink × intensity interactions were found for happy, sad, disgusted, or fearful expressions (ps > 0.059; see Figures 3(c)–(f)). Age was negatively associated with hostility ratings for angry, happy, and disgusted faces (ps < 0.035), while gender showed no significant effects (ps > 0.420). Trait anger was positively associated with hostile judgements of angry faces (*p* = 0.022), and AUDIT scores were negatively associated with hostile judgements of angry and disgusted faces (ps < 0.049).

### Approach/avoidance of emotional stimuli

**Figure 4.**
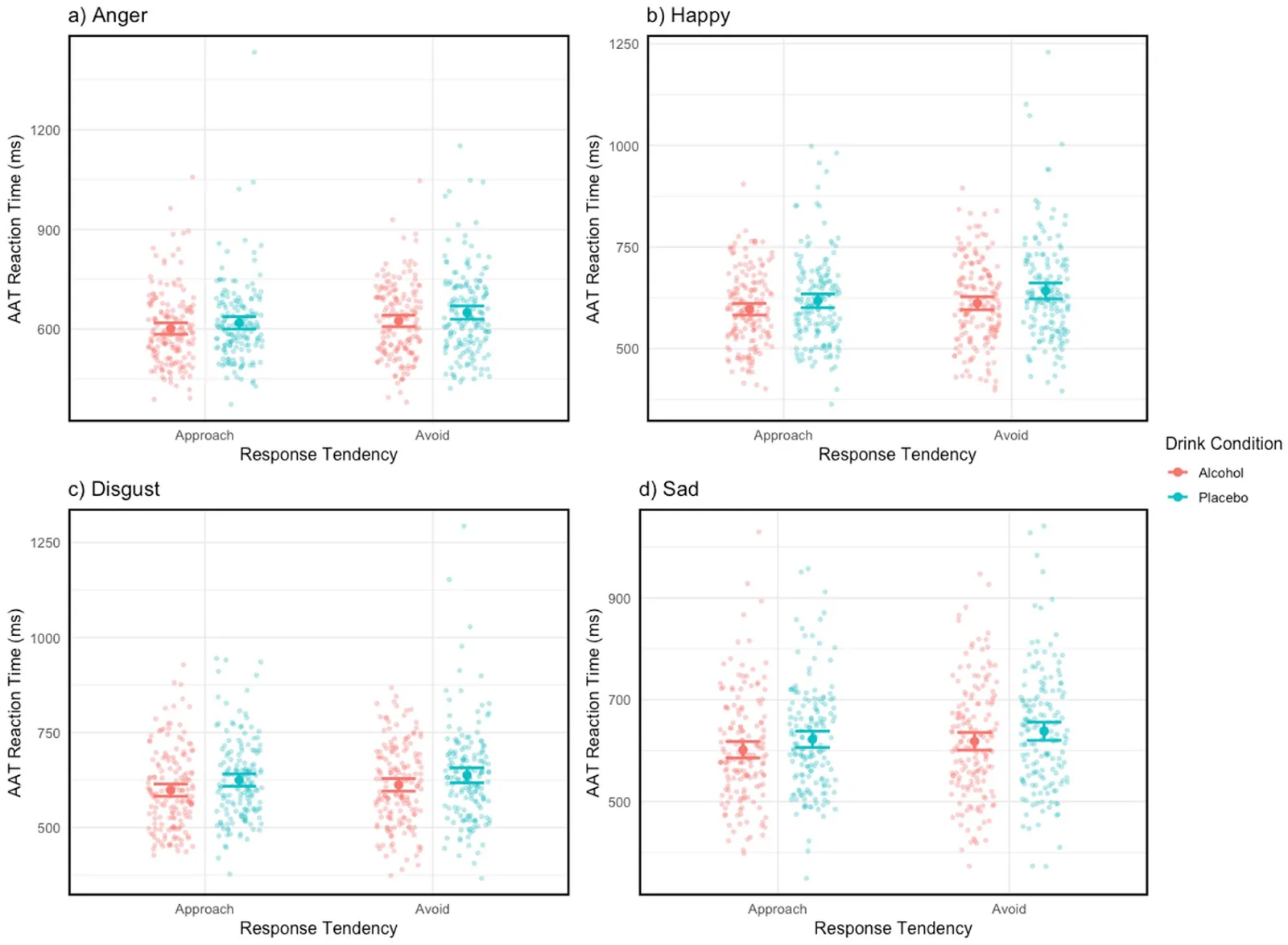
RT when cued to approach and avoid emotional facial stimuli (Angry, happy, sad, disgust) following alcohol and placebo drinks. Error bars represent 95% confidence intervals. RT: Reaction times.

Model estimates of approach/avoidance RT bias scores following alcohol and placebo for each emotion (Anger, Happy, Sad, Disgust) are shown in Figure 4(a) to (d), with full model estimates in Supplemental Table S2. The primary research objective was to test whether alcohol affects approach/avoidance tendencies when perceiving emotional facial expressions, when compared to placebo. There were significant main effects of drink condition for all emotions (ps < 0.007). RTs were faster following alcohol compared to placebo by an estimated 21.80 mseconds for angry expressions, 26.00 mseconds for happy, 20.60 mseconds for sad, and 26.00 mseconds for disgusted expressions. Significant main effects of approach/avoidance tendency were also observed for each emotion (ps < 0.002), with RTs faster in the approach-cued condition compared to the avoid-cued condition by 26.70 mseconds for angry expressions, 19.70 mseconds for happy, 16.40 mseconds for sad, and 13.20 mseconds for disgusted expressions. No significant drink × tendency interactions were observed for any emotion (ps > 0.260), indicating that the effect of alcohol on RTs did not differ between approach and avoidance conditions. Each model was adjusted for age, gender, trait anger, AUDIT score, face ethnicity, and AAT error rate. Males responded significantly faster than females across all emotional expressions (ps < 0.007), while no significant effects were found for age, trait anger, AUDIT score, face ethnicity, or error rate (ps > 0.290).

## Discussion

The primary aim of this study was to investigate whether social drinkers demonstrate greater HAB towards emotional facial expressions following alcohol compared to placebo. In addition, the influence alcohol has on approach avoidance tendencies when seeing emotional faces was also investigated. For global hostility ratings, there was no evidence of an effect of alcohol suggesting that social drinkers do not see emotions as more hostile following acute alcohol compared to placebo. However, when factoring emotional intensity, there was evidence to suggest that alcohol consumption influenced HAB of emotional expression as the intensity of the emotion increased. This interaction specifically highlighted that low intensity emotions (i.e. emotionally ambiguous to the perceiver) were rated as more hostile following alcohol. As the intensity of the emotion increased, this alcohol induced difference reduced, resulting in the difference between alcohol and placebo hostility ratings diminishing as a function of intensity.

Research suggests that ambiguous social information is often seen as more hostile (Milich and Dodge, 1984), and that alcohol consumption impairs global emotion processing (i.e. less recognition accuracy when intoxicated compared to sober) (Tucker and Vuchinich, 1983). It is likely that alcohol consumption leads to an increase in ambiguity of the emotion expressed by impairing the ability to accurately recognise the emotion, which results in greater HAB. Seeing ambiguous facial expressions as more hostile when intoxicated has social relevance as the propensity to see faces as more hostile may lead to increased aggression and violence (Wegrzyn et al., 2017). Similar past research indicates that higher levels of HAB are associated with increased aggression (Chen et al., 2012; Crick et al., 2002; Dodge, 2006) and consequently play a role in reactive aggressive behaviour (Crick and Dodge, 1996). Therefore, if individuals see an ambiguous facial expression as more hostile under the influence of alcohol, this may increase the likelihood of aggressive responding. These findings are also similar to those outlined in forensic populations (Schonenberg and Jusyte, 2014). Smeijers et al. (2017) similarly report a greater HAB towards facial stimuli in typically aggressive individuals (i.e. forensic outpatient sample) compared to healthy controls. This suggests that the hostile judgements of ambiguous emotions following acute alcohol consumption may follow a similar trend observed in typically aggressive individuals. Given the social context in which alcohol is often consumed, and the likelihood of alcohol increasing the ambiguity of facial expression recognition, HAB may be one mechanism by which alcohol consumption leads to aggressive behaviour in otherwise normal social drinkers.

At an emotion-specific level, happy facial expressions were seen as more hostile regardless of the emotional intensity displayed. As the happiness intensity increased, hostile judgements reduced; however, ambiguous (i.e. low intensity) and non-ambiguous (i.e. high intensity) displays of happiness were similarly perceived as more hostile following alcohol compared to placebo. Happy faces being seen as more hostile when intoxicated is of social importance as it is considered to be a positive emotion (Calvo and Beltran, 2013). Therefore, the increased hostile perception of happy faces may increase the likelihood of aggression by reducing the perceivers’’ exposure to positive cues that promote pro-social behaviour (Xu, 2025). Alcohol has been found to reduce orbitofrontal cortex (OFC) activity and heighten amygdala sensitivity to threat cues (Coccaro et al., 2007; Ohman, 2005), which may have led to biased negative interpretations. When OFC activity is compromised, the ability to integrate positive affective signals such as happy emotions is diminished, and the amygdala’s threat detection dominates. This is likely to create a HAB even for positive expressions like happy faces, reflecting a shift toward a hostile interpretation (Rule et al., 2002). The emotional intensity displayed in specific emotional faces was also important to consider as, previously mentioned, HAB often manifests when observations of social context cues are seen to be ambiguous (Milich and Dodge, 1984). As emotional intensity of angry and disgusted faces increased, so did perceived hostility suggesting that the clearer the display of these emotions the more hostile they were seen. On the other hand, when the intensity of happy and sad emotions increased, hostility ratings reduced. These findings support the notion that hostility interpretation is conceptually different to an anger perception bias (Deffenbacher, 2000; Eckhardt et al., 2004; Tsikandilakis et al., 2020) as the increased propensity to see hostility is not only limited to angry faces. There are a lot of facial characteristic similarities between an angry face and a disgusted one displayed in isolation (Wieser and Brosch, 2012), which may explain why both expressions were similarly rated as more hostile as intensity increased. When considering the drink by intensity interaction for these emotions, angry faces were rated as more hostile at low emotional intensities (i.e. ambiguous displays of anger) following alcohol compared to placebo. This drink-related difference reduced as intensity increased (i.e. the increased hostility rating associated with alcohol diminished as the emotion displayed in the expressions became clearer). So, whilst ambiguous displays of anger were rated as more hostile following alcohol compared to placebo, high-intensity displays of this emotion (i.e. unambiguous) were not. The impairing effects of alcohol on the OFC and amygdala connectivity (Coccaro et al., 2007; Ohman, 2005) are likely also implicated in this interaction effect. A reduction of OFC activity following alcohol limits top-down regulation of the amygdala resulting in heightened sensitivity to subtle anger cues (Attwood and Munafò, 2014). Consequently, ambiguous angry faces may be perceived as more hostile following alcohol because interpretation relies more on automatic threat processing than contextual facial cues. At higher emotional intensities, these effects of alcohol diminish.

Clear comparisons and meta-inferences can be drawn from past research exploring the effects of alcohol on emotional face processing and the present study testing HAB towards these stimuli. Our previous work found that acute alcohol consumption impairs emotion recognition accuracy, increasing the ambiguity of facial expressions (Eastwood et al., 2020). The present study extends this by showing that alcohol heightens HAB for ambiguous emotional expressions, with this bias diminishing as emotional intensity increases. Together, these findings suggest that alcohol may lead social drinkers to infer hostile intent through two complementary behavioural effects: impaired emotion recognition and increased HAB. These outcomes are known correlates of aggression, underscoring the importance of understanding alcohol’s impact on socio-emotional processing (Chen et al., 2012; Crick et al., 2002; Dodge, 2006). Pharmacologically, alcohol’s effects on GABAergic and glutamatergic neurotransmission may dampen cortical processing and emotional salience detection, leading to both reduced recognition accuracy and biased interpretations (Dharavath et al., 2023; Roberto et al., 2012). Specifically, the OFC, amygdala and their connectivity have been identified as key factors involved in regulating emotional responses and supporting accurate interpretation of facial cues (Davidson et al., 2000; Rolls et al., 2006). Acute alcohol consumption compromises this by reducing OFC activity and its functional connectivity with the amygdala (Gorka et al., 2013), which may explain alcohol induced emotional face recognition deficits (Eastwood et al., 2020) and the HABs towards ambiguous faces.

This study also hypothesised that social drinkers would display a greater tendency to approach a hostile stimulus (i.e. angry and disgusted faces) following alcohol compared to placebo. Findings indicate that this was not the case. There was evidence for a main effect of drink, suggesting faster RTs following alcohol compared to placebo. There was also evidence for a main effect of tendency suggesting a faster RT in approach compared to avoidance conditions for all emotional expression (anger, happy, sad, disgust), but this was not moderated by alcohol consumption (no evidence of an approach/avoidance tendency by drink interaction). This suggests that alcohol consumption generally makes people quicker on the AAT task (qualified by the main effects of drink on RTs), and that individuals are faster to approach than avoid emotional stimuli (qualified by the main effects of tendency on RTs). But social drinkers show no increased/decreased tendency to approach or avoid an angry or disgusted face following alcohol compared to placebo. Similarly, for happy and sad faces, there was no evidence to suggest that the propensity to approach or avoid was moderated by acute alcohol consumption. These findings may be due to the nature of the approach/avoidance task used in the present study. Implicit instructions meant that participants were not required to attend to the emotional valence of the stimuli but were instead required to respond to a task-irrelevant feature (i.e. the border of the image displaying the emotional facial expression). Feedback was given on each trial in the form of image zooming. For approach trials, this meant that images would increase in size and in avoidance trials they would decrease (Phaf et al., 2014). Effects established using this implicit version would suggest an automatic link between affective information processing (i.e. emotional valence) and approach/avoidance tendencies. As there was no evidence of a difference in the tendency to approach or avoid emotional stimuli (angry, happy, sad and disgusted faces), these findings suggest that behavioural motivation may not be an automatic response to these social cues. Previous research has shown that the implicit version of the AAT tends to result in lower reliability (Krieglmeyer and Deutsch, 2010), which may have also influenced the null findings. The internal reliability of the AAT used in the present study was assessed using a bootstrapped split-half analysis (Pronk et al., 2022; Warrens, 2016). The resulting reliability coefficient was negative (*r* = −0.32, *p* = .004) indicating poor internal consistency. This means that participants’ responses were not reliable across different parts of the task, making it difficult to confidently interpret the results as reflecting true approach or avoidance tendencies. A review evaluating implicit versus explicit instructions in approach avoidance paradigms concludes that explicit instructions often yield bigger effects sizes (Krieglmeyer and Deutsch, 2010). In this version of the task, participants are required to explicitly evaluate the emotional valence of a stimuli by responding with an approach action (i.e. joystick pull) or avoidance action (i.e. joystick push) when seeing positive and negative stimuli, respectively. It may be that approach/avoidance tendencies under the influence of alcohol require the individual to make conscious evaluations of cues in order to judge whether the social information should be approached or avoided. Future research should investigate implicit versus explicit instruction on approach/avoidance tendencies following alcohol to investigate whether processing of social stimuli is automatic or driven by the conscious evaluation of valence.

Although the study shows meaningful insights into alcohol’s effects on hostile judgements of emotional faces, certain limitations may have influenced the interpretation of these findings. Emotional face processing and HABs are not solely driven by facial features in isolation. Rather, they are shaped by the broader interpersonal and situational context (Bublatzky et al., 2014; Wieser and Brosch, 2012). By presenting facial stimuli in isolation, these dynamic and interactive elements of real-world social cognition may not be captured. Future research should aim to incorporate contextual variables such as background scenes, social roles, or relational information to better understand how alcohol modulates emotional face processing and hostile attribution in ecologically valid settings (Calbi et al., 2017). Further limitations surround methodological and design approaches. Alcohol dosing was not adjusted for sex-based physiological differences. Although body weight was considered in calculating alcohol dosage, it is well-established that women generally exhibit higher blood alcohol concentrations than men after consuming equivalent doses due to differences in body composition (Vatsalya et al., 2023); specifically, a higher proportion of body fat (Baraona et al., 2001) and lower total body water (Goist and Sutker, 1985). These differences may have led to greater intoxication effects in female participants, potentially influencing their emotional processing and behavioural responses. As a result, the observed effects may partially reflect differential alcohol metabolism rather than uniform pharmacological effects, limiting the generalisability of the findings across sexes. Future research should adjust alcohol dosing based on sex-specific physiological differences to ensure more accurate comparisons across sexes. Another limitation of the present study is that the placebo manipulation was not fully effective. Despite efforts to mask the presence or absence of alcohol, such as informing participants they would receive a drink that may or may not contain alcohol and applying alcohol to the rim of the glass, most participants correctly identified which session involved alcohol. It is well established that the expectation of alcohol influences how individuals respond (Testa et al., 2006). This suggests that expectancy effects may have influenced participants’ responses, making it difficult to isolate the pharmacological effects of alcohol from psychological expectations. Future studies should improve placebo controls by using more sophisticated masking techniques or alternative methods to better blind participants regarding their consumption status. Further limitations relate to sample characteristics. The recruited participants varied widely in their self-reported alcohol consumption, ranging from 5 to 35 drinks per week. This heterogeneity may have influenced individual differences in alcohol tolerance and metabolic biotransformation, complicating interpretation of the results (Würtz et al., 2016). Additionally, the sample included a broad weight range, and obesity is known to affect alcohol distribution and metabolism (Berton et al., 2023; Knibbe et al., 2015). Future research should consider stratified sampling based on alcohol use and consideration of metabolic health factors such as obesity to clarify individual variability in alcohol’s impact on emotional processing and hostile judgements.

## Conclusion

Our findings suggest that ambiguous emotional facial expressions are judged as more hostile following acute alcohol consumption compared to placebo. This global HAB when seeing ambiguous faces may increase the likelihood of maladaptive behaviour, as the greater propensity to judge socially relevant stimuli as hostile has been shown to increase aggressive responding. At an emotion-specific level, happy faces are seen as more hostile following alcohol. Happy is considered to be a positive emotion. If alcohol induces a HAB of this emotion, positive social cues may be missed, which may decrease pro-social behaviour and increase the likelihood of aggressive responding. This study failed to find an effect of alcohol on approach/avoidance tendencies when seeing emotional facial expression. These effects may be more pronounced in typically aggressive populations (i.e. forensic samples), and future work could address whether these specific groups demonstrate different approach/avoidance tendencies following alcohol. More work is required to establish the behavioural motivations associated with hostile and non-hostile faces. This could involve investigating the precise context in which hostile displays of emotion become approached.

## Supplemental Material

sj-docx-1-jop-10.1177_02698811251408743 – Supplemental material for Effects of acute alcohol consumption on hostile attribution bias towards emotional facial expressions and implicit approach/avoidance tendencies Supplemental material, sj-docx-1-jop-10.1177_02698811251408743 for Effects of acute alcohol consumption on hostile attribution bias towards emotional facial expressions and implicit approach/avoidance tendencies by Andrew P. R. Eastwood, Ian S. Penton-Voak, Marcus R. Munafò and Angela S. Attwood in Journal of Psychopharmacology
