# Supplementary material for: Effects of acute alcohol consumption on hostile attribution bias towards emotional facial expressions and implicit approach/avoidance tendencies
Source: J Psychopharmacol. 2026 Mar 23;40(9):1392–404. doi: 10.1177/02698811251408743 (PMC13490694; doi:10.1177/02698811251408743)
Supplement: Supplementary file 1 [file sj-docx-1-jop-10.1177_02698811251408743.docx]

Supplementary Materials

# Supplementary Text S1: Protocol Analyses

**HABT Analysis**

The Hostile Attribution Bias Task (HABT) was analysed using the percentage of hostile responses as the primary outcome measure. Outliers were identified using boxplots, and participant data were excluded if scores exceeded 1.5 times the interquartile range. Normality was assessed via skewness and kurtosis statistics. Where sphericity was violated Greenhouse-Geisser corrections were applied. A 2 (drink: alcohol, placebo) × 6 (emotion: happy, sad, angry, surprise, fear, disgust) repeated measures ANOVA was conducted to examine the effect of alcohol on hostile attributions toward emotional expressions.

A repeated measures ANOVA showed a significant main effect of Emotion on HAB scores, F(1.87, 153.74) = 497.83, p < .001, η²_G_ = .729 (Greenhouse-Geisser correction), indicating substantial variation in approach-avoidance tendencies across emotional expressions. Specifically, HAB scores were highest for Anger (M = 72%, SD = 17%) and Disgust (M = 62%, SD = 30%), suggesting strong approach tendencies in response to these emotions. In contrast, scores were markedly lower for Fearful (M = 4%, SD = 12%), Happy (M = 7%, SD = 12%), and Sad (M = 9%, SD = 14%) faces, indicating greater avoidance or neutral responses. Post hoc pairwise comparisons using Tukey’s HSD revealed that HAB scores were significantly higher for Anger and Disgust compared to all other emotions (ps < .001). No significant differences were found between Happy and Sad, Happy and Fearful, Sad and Fearful, or Anger and Disgust (*ps* > .458). The main effect of Drink and the Drink × Emotion interaction were not significant (*ps* > .323).

**AAT Analysis**
The Approach-Avoidance Task (AAT) was analysed using reaction time (RT) bias scores (Avoid RT – Approach RT) and error rates as outcome measures. Positive RT bias scores indicate a greater preference for approach responses and negative scores indicate a greater preference for avoid responses. Error rates were used to screen for outliers, and 3 participants were removed from the analysis as rates were relatively high (3 standard deviations from the mean). Further inspection of these data suggests that the participants misunderstood the task (i.e., high errors scores indicated using the task response options the wrong way around). A 2 (drink: alcohol, placebo) × 2 (ethnicity: white, black) × 4 (emotion: happy, angry, sad, disgust) repeated measures ANOVA was used to evaluate the influence of alcohol on approach/avoidance tendencies (N=81). Error rates were analysed with an additional within-subjects factor of response tendency (approach, avoidance).

A repeated measures ANOVA was conducted to examine the effects of drink condition, face ethnicity, and emotional expression on approach/avoidance tendencies, as measured by a RT bias score. The analysis revealed a significant main effect of Ethnicity, *F(1, 80) = 11.96, p < .001, η²₉ = .005*, indicating that participants approached black faces (M =25.09ms, SD = 81.62ms) quicker than white (M = 12.86ms, SD = 84.64ms; effect size was small). No other main effects or interactions were statistically significant (all *ps* > .130). A 2 (Drink: alcohol, placebo) × 2 (Ethnicity: White, Black) × 4 (Emotion: happy, angry, sad, disgust) × 2 (Tendency: approach, avoidance) repeated measures ANOVA was conducted to examine the effects of these factors on error rates in the AAT. There was a significant main effect of Drink, *F*(1, 80) = 6.50, *p* = .013, η²_G_ = .012, indicating that more errors were made following alcohol (M = .014, SD = .050) when compared to placebo (M = .005, SD = .028) on the AAT. No other main effects or interactions were statistically significant (*ps* > .05).

| Supplementary Table S1: LME model main effect estimates, 95 % Confidence intervals, and p-values for global hostile ratings of emotional faces, as well as for each specific emotion (Angry, Happy, Sad, Disgust, Fear). Random effect variance for subject ID and Drink. | | | | | | | | | | | | | | | | | | |
| --- | --- | --- | --- | --- | --- | --- | --- | --- | --- | --- | --- | --- | --- | --- | --- | --- | --- | --- |
| **Fixed Effects** | | | | | | | | | | | | | | | | | | |
|  | **Global** | | | **Angry** | | | **Happy** | | | **Sad** | | | **Disgust** | | | **Fear** | | |
| *Predictors* | *Estimate* | *95% CI* | *p* | *Estimate* | *95% CI* | *p* | *Estimate* | *95% CI* | *p* | *Estimate* | *95% CI* | *p* | *Estimate* | *95% CI* | *p* | *Estimate* | *95% CI* | *p* |
| **Block 1: Main Effects (Intensity + Drink)** | | | | | | | | | | | | | | | | | | |
| *(Intercept)* | 43.45 | 27.70 – 59.19 | **<0.001** | 50.960 | 31.806 – 70.115 | **<0.001** | 18.627 | 4.656 – 32.599 | **0.010** | 23.530 | 2.676 – 44.383 | **0.027** | 115.640 | 79.525 – 151.755 | **<0.001** | 10.913 | -7.204 – 29.030 | 0.234 |
| Age | -0.89 | -1.46 – -0.33 | **0.002** | -1.382 | -2.067 – -0.697 | **<0.001** | -0.541 | -1.043 – -0.040 | **0.035** | -0.402 | -1.150 – 0.346 | 0.288 | -2.292 | -3.589 – -0.995 | **0.001** | -0.076 | -0.719 – 0.566 | 0.814 |
| Gender [Female ^R^, Male] | -1.46 | -6.47 – 3.55 | 0.564 | -1.567 | -7.635 – 4.501 | 0.609 | 1.196 | -3.244 – 5.635 | 0.593 | -2.693 | -9.318 – 3.933 | 0.421 | -3.952 | -15.438 – 7.534 | 0.495 | -1.794 | -7.487 – 3.898 | 0.532 |
| Trait Anger | 0.03 | -0.65 – 0.72 | 0.919 | 0.972 | 0.141 – 1.803 | **0.022** | 0.373 | -0.235 – 0.981 | 0.225 | -0.017 | -0.924 – 0.890 | 0.971 | -0.859 | -2.432 – 0.713 | 0.280 | -0.065 | -0.845 – 0.714 | 0.868 |
| AUDIT | -0.54 | -1.05 – -0.04 | **0.036** | -0.613 | -1.224 – -0.002 | **0.049** | -0.324 | -0.771 – 0.124 | 0.154 | -0.214 | -0.882 – 0.453 | 0.525 | -1.397 | -2.555 – -0.240 | **0.019** | -0.134 | -0.708 – 0.439 | 0.642 |
| Intensity | 1.61 | 1.51 – 1.71 | **<0.001** | 5.513 | 5.264 – 5.762 | **<0.001** | -0.561 | -0.705 – -0.417 | **<0.001** | -0.242 | -0.412 – -0.071 | **0.006** | 3.297 | 3.052 – 3.542 | **<0.001** | 0.017 | -0.083 – 0.116 | 0.741 |
| Drink [Placebo ^R^, Alcohol] | 1.06 | -1.15 – 3.28 | 0.342 | -0.360 | -3.620 – 2.900 | 0.827 | 3.013 | 0.759 – 5.268 | **0.009** | 1.388 | -1.539 – 4.316 | 0.348 | 2.937 | -1.698 – 7.571 | 0.211 | -1.536 | -4.957 – 1.885 | 0.375 |
| **Block 2: Interaction Term (Intensity * Drink)** | | | | | | | | | | | | | | | | | | |
| *(Intercept)* | 42.24 | 26.48 – 58.00 | **<0.001** | 48.590 | 29.339 – 67.841 | **<0.001** | 18.316 | 4.298 – 32.334 | **0.011** | 22.756 | 1.860 – 43.653 | **0.033** | 113.752 | 77.585 – 149.919 | **<0.001** | 10.299 | -7.836 – 28.433 | 0.262 |
| Intensity | 1.76 | 1.62 – 1.90 | **<0.001** | 5.810 | 5.459 – 6.162 | **<0.001** | -0.522 | -0.726 – -0.318 | **<0.001** | -0.145 | -0.387 – 0.097 | 0.240 | 3.533 | 3.187 – 3.880 | **<0.001** | 0.094 | -0.047 – 0.234 | 0.193 |
| Drink [Placebo ^R^, Alcohol] | 3.48 | 0.78 – 6.19 | **0.012** | 4.399 | -0.725 – 9.524 | 0.092 | 3.635 | 0.423 – 6.847 | **0.027** | 2.932 | -1.058 – 6.922 | 0.149 | 6.712 | 0.665 – 12.759 | **0.030** | -0.310 | -4.074 – 3.454 | 0.871 |
| Intensity * Drink [Placebo ^R^, Alcohol] | -0.30 | -0.50 – -0.11 | **0.002** | -0.595 | -1.092 – -0.098 | **0.019** | -0.078 | -0.366 – 0.210 | 0.597 | -0.193 | -0.535 – 0.149 | 0.268 | -0.472 | -0.962 – 0.018 | 0.059 | -0.153 | -0.352 – 0.046 | 0.131 |
| *Table continues on next page.* | | | | | | | | | | | | | | | | | | |

|  | **Global** | | **Angry** | | **Happy** | | **Sad** | | **Disgust** | | **Fear** | |
| --- | --- | --- | --- | --- | --- | --- | --- | --- | --- | --- | --- | --- |
| **Random Effects** | | | | | | | | | | | | |
|  | *Variance* | *SD* | *Variance* | *SD* | *Variance* | *SD* | *Variance* | *SD* | *Variance* | *SD* | *Variance* | *SD* |
| Residual | 117.29 | 10.830 | 754.9 | 27.48 | 253.29 | 15.915 | 356.4 | 18.88 | 734.5 | 27.10 | 120.8 | 10.99 |
| ID | 128.27 | 11.326 | 151.2 | 12.30 | 82.63 | 9.090 | 223.2 | 14.94 | 671.0 | 25.90 | 352.6 | 18.78 |
| Drink [Placebo, Alcohol] | 88.71 | 9.418 | 124.9 | 11.18 | 74.08 | 8.607 | 134.4 | 11.59 | 358.1 | 18.92 | 232.4 | 15.24 |
| ICC | 0.54 | | 0.20 | | 0.31 | | 0.40 | | 0.49 | | 0.68 | |
| N | 84 _ID_ | | 84 _ID_ | | 84 _ID_ | | 84 _ID_ | | 84 _ID_ | | 84 _ID_ | |
| Observations | 2520 | | 2517 | | 2517 | | 2517 | | 2520 | | 2512 | |
| Marginal R^2^ / Conditional R^2^ | 0.218 / 0.644 | | 0.395 / 0.517 | | 0.043 / 0.340 | | 0.014 / 0.404 | | 0.213 / 0.598 | | 0.007 / 0.677 | |
| *Note: Age in years, Gender (Female, Male), Trait Anger Subscale of the STAXi-2, AUDIT Sum, Intensity of emotion (1 – emotionally ambiguous – 15 full example of emotion), Drink (Placebo, Alcohol). LME models were used to explore global as well as emotion specific hostile attribution bias. In Block 1, the fixed effects of drink and intensity were entered into each linear mixed effect model to test the main effects of drink and intensity. In Block 2, a drink by intensity interaction term was entered into each model. Age, gender, trait anger and AUDIT were also entered into each model to control for their influence. Random effects for each full model included random intercepts for subject ID and by ID random slopes for the effect of drink. P values for each fixed effect were estimated using Kenward-Rogers d.f.*  *^R^ Reference level for each categorical variable.* | | | | | | | | | | | | |

| Supplementary Table S2: LME model estimates, 95 % Confidence intervals, and p-values for approach/avoidance RT scores for each specific emotional facial expression (Angry, Happy, Sad, Disgust, Fear). Random effect variance for subject ID and Drink. | | | | | | | | | | | | |
| --- | --- | --- | --- | --- | --- | --- | --- | --- | --- | --- | --- | --- |
| **Fixed Effects** | | | | | | | | | | | | |
|  | **Anger** | | | **Happy** | | | **Sad** | | | **Disgust** | | |
| *Predictors* | *Estimates* | *95% CI* | *p* | *Estimates* | *95% CI* | *p* | *Estimates* | *95% CI* | *p* | *Estimates* | *95% CI* | *p* |
| **Block 1: Main Effects (Tendency + Drink)** | | | | | | | | | | | | |
| *(Intercept)* | 685.307 | 542.796 – 827.819 | **<0.001** | 675.494 | 545.993 – 804.995 | **<0.001** | 669.927 | 533.348 – 806.506 | **<0.001** | 684.945 | 545.912 – 823.978 | **<0.001** |
| Age | -0.793 | -6.184 – 4.599 | 0.770 | 0.331 | -4.557 – 5.219 | 0.893 | 0.156 | -5.016 – 5.327 | 0.952 | -0.330 | -5.591 – 4.931 | 0.901 |
| Gender [Female ^R^, Male] | -61.118 | -105.226 – -17.010 | **0.007** | -53.081 | -93.046 – -13.116 | **0.010** | -52.461 | -94.759 – -10.162 | **0.016** | -56.529 | -99.567 – -13.491 | **0.011** |
| Trait Anger | -0.159 | -6.121 – 5.804 | 0.958 | -1.466 | -6.869 – 3.936 | 0.590 | -0.449 | -6.166 – 5.268 | 0.876 | -1.294 | -7.111 – 4.523 | 0.659 |
| AUDIT | -1.274 | -5.704 – 3.156 | 0.568 | -1.525 | -5.539 – 2.488 | 0.451 | -1.574 | -5.822 – 2.674 | 0.463 | -0.511 | -4.834 – 3.812 | 0.814 |
| Ethnicity [Black ^R^, White] | -1.393 | -11.337 – 8.550 | 0.783 | 4.379 | -3.874 – 12.631 | 0.298 | -3.607 | -11.916 – 4.702 | 0.394 | 1.176 | -6.959 – 9.312 | 0.776 |
| Error Rate | 39.286 | -120.225 – 198.798 | 0.629 | 54.941 | -76.785 – 186.667 | 0.413 | 38.202 | -86.653 – 163.056 | 0.548 | 57.544 | -87.899 – 202.988 | 0.437 |
| Tendency [Approach ^R^, Avoid] | 26.679 | 16.723 – 36.634 | **<0.001** | 19.737 | 11.476 – 27.998 | **<0.001** | 16.377 | 8.073 – 24.681 | **<0.001** | 13.161 | 5.043 – 21.278 | **0.002** |
| Drink [Placebo ^R^, Alcohol] | -21.755 | -37.493 – -6.016 | **0.007** | -26.038 | -42.815 – -9.262 | **0.003** | -20.641 | -35.166 – -6.116 | **0.006** | -26.013 | -41.371 – -10.656 | **0.001** |
| **Block 2: Interaction Term (Tendency * Drink)** | | | | | | | | | | | | |
| *(Intercept)* | 683.219 | 540.610 – 825.827 | **<0.001** | 673.211 | 543.653 – 802.769 | **<0.001** | 670.134 | 533.499 – 806.770 | **<0.001** | 685.374 | 546.287 – 824.460 | **<0.001** |
| Tendency [Approach ^R^, Avoid] | 30.756 | 16.699 – 44.813 | **<0.001** | 24.390 | 12.726 – 36.054 | **<0.001** | 15.956 | 4.212 – 27.699 | **0.008** | 12.292 | 0.788 – 23.795 | **0.036** |
| Drink [Placebo ^R^, Alcohol] | -17.705 | -36.207 – 0.797 | 0.061 | -21.354 | -40.015 – -2.693 | **0.025** | -21.066 | -37.766 – -4.366 | **0.014** | -26.883 | -44.213 – -9.553 | **0.003** |
| Tendency [Approach ^R^, Avoid] * Drink [Placebo ^R^, Alcohol] | -8.186 | -28.104 – 11.732 | 0.420 | -9.321 | -25.822 – 7.179 | 0.268 | 0.845 | -15.772 – 17.462 | 0.920 | 1.735 | -14.525 – 17.995 | 0.834 |
| *Table continues on next page* | | | | | | | | | | | | |

|  | **Anger** | | **Happy** | | **Sad** | | **Disgust** | |
| --- | --- | --- | --- | --- | --- | --- | --- | --- |
| **Random Effects** | | | | | | | | |
|  | *Variance* | *SD* | *Variance* | *SD* | *Variance* | *SD* | *Variance* | *SD* |
| Residual | 4146 | 64.39 | 2853 | 53.42 | 2893 | 53.79 | 2767 | 52.60 |
| ID | 10724 | 103.56 | 11036 | 105.05 | 9597 | 97.97 | 10522 | 102.58 |
| Drink [Placebo=0, Alcohol=1] | 2935 | 54.17 | 4292 | 65.52 | 2832 | 53.21 | 3436 | 58.62 |
| ICC | 0.70 | | 0.76 | | 0.75 | | 0.77 | |
| N | 81 _ID_ | | 81 _ID_ | | 81 _ID_ | | 81 _ID_ | |
| Observations | 648 | | 648 | | 648 | | 648 | |
| Marginal R^2^ / Conditional R^2^ | 0.091 / 0.724 | | 0.092 / 0.780 | | 0.079 / 0.772 | | 0.086 / 0.791 | |
| *Note: Age in years, Gender (Female, Male), Trait Anger Subscale of the STAXi-2, AUDIT Sum, Ethnicity of facial expressions (Black, White), Error Rate is the proportion of mistakes made (i.e., responded with approach in avoidance cued trials and vice versa), Tendency (Approach, Avoid), Drink (Placebo, Alcohol). In Block 1, the fixed effects of tendency and drink were entered into a linear mixed effects model to test the main effects of tendency and drink on RT. In Block 2, a tendency by drink interaction term was entered into the model to test the interaction between tendency and drink on RT. Age, gender, trait anger AUDIT, error rate were also entered into each model to control for their influence. Random effects for each full model included random intercepts for subject ID and by ID random slopes for the effect of drink. P values for each fixed effect were estimated using Kenward-Rogers d.f.*  *^R^ Reference level for each categorical variable* | | | | | | | | |
